# Editorial: Methods in cancer imaging and image-directed interventions

**DOI:** 10.3389/fonc.2023.1293020

**Published:** 2023-10-17

**Authors:** Jasper Nijkamp, Bahram Mohajer

**Affiliations:** ^1^ Department of Clinical Medicine, Aarhus University, Aarhus, Denmark; ^2^ Danish Center for Particle Therapy, Aarhus University Hospital, Aarhus, Denmark; ^3^ Russell H. Morgan Department of Radiology and Radiological Science, Johns Hopkins University School of Medicine, Baltimore, MD, United States

**Keywords:** methods, cancer imaging, image-directed interventions, image-guidance, radiotherapy, radiology, radiomics

## Introduction

The methods we employ to visualize, analyze, and intervene in cancerous tissues rapidly evolve. These methods, which include various imaging modalities and image-guided therapies, play a vital role in the accurate diagnosis, precise treatment planning, and targeted interventions for cancer patients.

We are glad to see a total of 16 high-quality papers published in this Research Topic, of which twelve original articles, three reviews, and one methods paper, from various countries and regions. As can be expected from the broad topic, a wide range of papers is presented.

## Molecular, magnetic resonance, and ultrasound imaging

For molecular imaging, Liang et al. presented a novel molecular probe for imaging programmed death ligand 1 (PD-L1) with single-photon emission computed tomography (SPECT). Their PD-L1 targeting affibody with ^99m^Tc labelling showed high *in vivo* affinity and quick blood clearance.

Lin et al. performed a prospective study comparing ^18^FDG-PET/CT with ^68^Ga-FAPI-04 PET/CT on colorectal cancer staging. Differences in staging between both scans were investigated in 61 patients. FAPI-PET led to TNM upstaging in 10, and downstaging in 5 patients, which consecutively altered the treatment course in 13 patients, primarily due to better nodal and metastasis detection.

Three studies assessed use of MRI in tumor grading and tissue characterization. Apparent diffusion coefficient (ADC) is often suggested as biomarker of prostate tumor aggressiveness. Bengtsson et al. addressed the challenges in generalizing ADC cut-off values between scanners and centers with regard to tumor aggressiveness. They showed that ADC (and its ratios) did not correlate with tumor Gleason Grade, and should therefore be used with caution as biomarker.

Secondly, Waqar et al. focused their narrative review on current status of habitat MR-imaging in glioblastoma. With habitat imaging, tumors can be subdivided into regions that express similar imaging characteristics, assuming that these regions represent different tumor biology. They provide an overview of the current work, challenges of producing relevant and reproducible habitats, and clinical applications for personalized treatment.

Finally, Micek et al. reviewed the current applications of non-contrast-enhanced quantitative MRI parameters, specifically T1 and T2 relaxation time, for tissue characterization. They focused on epithelium in breast cancer, but also reviewed studies on lung, prostate, skin, kidney, and liver malignancies. They concluded that in most studies only T1 or T2 relaxation times were reported, resulting in limited groundwork to the value of combining the two in differentiating tissues.

In another study, Zhang et al. show the diagnostic value of virtual touch tissue quantification (VTQ) ultrasonography in combination with pleural effusion chemokine expression quantification (CXCL13) for detection of malignant lung nodules. They show a synergistic diagnostic potential of CXCL13 and VTQ for differentiating malignant and benign pleural effusions.

## Image segmentation and validation

Ren et al. provide a novel prostate and prostate lesion segmentation model, based on UNet, but using dense blocks, a convolution block attention module, and group norm-Atrous spatial pyramidal pooling. Their input data was DWI imaging only, and they show that their approach outperforms other well-known architectures.

Sahlsten et al. investigated the effect of defacing CT and MRI scans on segmentation accuracy of organs at risk in the head and neck area. Defacing imaging data without affecting the segmentation accuracy could help to overcome challenges in data sharing. Of the eight available defacing models, only 3 performed acceptably on MRI, none did well on CT. Segmentation accuracy was influenced by the defacing tools, and improvements are needed to assure safe data sharing.

With an innovative design and with the aim of correlating imaging findings with histology in ovarian tumors, Delgado-Ortet et al. presented a method for designing and 3D printing molds. The work is inspired by similar workflows presented in imaging validation of prostate cancer but applied in the challenging setting of ovarian tumors.

## Response assessment, radiomics, and prediction models

Iannessi and Beaumont evaluated the effect of observer variation in standardized response assessment (RECIST) in clinical trials of patient with lung cancer with blinded independent central review. They show that even with standardization, there are substantial variabilities at baseline and provide evidence of observer dependent reading patterns.

In the field of radiomics, Liu et al. provide an bibliometric overview of the research that is being conducted in the area of FDG-PET/CT radiomics. They included 361 original articles and 96 reviews, and provide which countries, institutes, and individuals are the drivers of the research in this area.

In a diagnostic application, Cheng et al. developed an MRI radiomics model to differentiate between ovarian sex cord-stromal tumors and epithelial ovarian cancers. They showed best performance when combining clinical characteristics, conventional MRI parameters (e.g. ADC), and a radiomics score based on 9 features. For ease of use, they also developed a nomogram.

Huang et al. aimed to predict response to radiofrequency ablation (RFA) of colorectal lung metastasis using radiomics. Their model contained combinations of CT-radiomics features of the pre-RFA metastasis and the ablation zone, plus cancer antigen 19-9 and location of the metastasis as clinical features. With an accuracy of 82.6%, this work shows potential to guide personalized follow-up in these patients.

In another study, using multiparametric (mp)MRI, without radiomics, Meng et al. provided an approach to predict the surgical resection margins after laparoscopic prostatectomy. This approach could help choosing optimal treatment for prostate cancer patients. They presented nomograms based on clinical parameters, mpMRI, and a combination, with the latter as most accurate (AUC=0.756).

## Radiotherapy

In the field of radiotherapy, McDonald et al. provided a comprehensive review of dose accumulation for MR-guided adaptive radiotherapy. They cover the whole range from practical considerations to state-of-the-art clinical implementation.

Finally, Nankali et al. describe a method to monitor intrafraction tumor motion and accumulate the fraction dose during liver pencil beam scanning proton therapy. The illustration of the method on three patient cases is impressive, but the pathway to broad clinical implementation is challenged by integration needs.

This Research Topic provides a cross-sectional snapshot of a rapidly evolving field. We are excited to observe where the field progresses in coming years.

## Author contributions

JN: Writing – original draft, Writing – review & editing. BM: Writing – original draft, Writing – review & editing.

